# Orthostatic Cerebral Hypoperfusion Syndrome

**DOI:** 10.3389/fnagi.2016.00022

**Published:** 2016-02-16

**Authors:** Peter Novak

**Affiliations:** ^1^Department of Neurology, Brigham and Women’s Faulkner Hospital, Harvard Medical School, Boston, MA, USA

**Keywords:** OCHOs, POTS, OH, orthostatic, hypoperfusion, hypotension, QASAT

## Abstract

**Objective:**

Orthostatic dizziness without orthostatic hypotension is common but underlying pathophysiology is poorly understood. This study describes orthostatic cerebral hypoperfusion syndrome (OCHOs). OCHOs is defined by (1) abnormal orthostatic drop of cerebral blood flow velocity (CBFv) during the tilt test and (2) absence of orthostatic hypotension, arrhythmia, vascular abnormalities, or other causes of abnormal orthostatic CBFv.

**Methods:**

This retrospective study included patients referred for evaluation of unexplained orthostatic dizziness. Patients underwent standardized autonomic testing, including 10 min of tilt test. The following signals were monitored: heart rate, end tidal CO_2_, blood pressure, and CBFv from the middle cerebral artery using transcranial Doppler. Patients were screened for OCHOs. Patients who fulfilled the OCHOs criteria were compared to age- and gender-matched controls.

**Results:**

From 1279 screened patients, 102 patients (60/42 women/men, age 51.1 ± 14.9, range 19–84 years) fulfilled criteria of OCHOs. There was no difference in baseline supine hemodynamic variables between OCHOs and the control group. During the tilt, mean CBFv decreased 24.1 ± 8.2% in OCHOs versus 4.2 ± 5.6% in controls (*p* < 0.0001) without orthostatic hypotension in both groups. Supine mean blood pressure (OCHOs/controls, 90.5 ± 10.6/91.1 ± 9.4 mmHg, *p* = 0.62) remained unchanged during the tilt (90.4 ± 9.7/92.1 ± 9.6 mmHg, *p* = 0.2). End tidal CO_2_ and heart rate responses to the tilt were normal and equal in both groups.

**Conclusion:**

OCHOs is a novel syndrome of low orthostatic CBFv. Two main pathophysiological mechanisms are proposed, including active cerebral vasoconstriction and passive increase of peripheral venous compliance. OCHOs may be a common cause of orthostatic dizziness.

## Introduction

Orthostatic dizziness is common, affecting 2–19% of elderly (Ensrud et al., 1992; Hoffman et al., 1999; Wu et al., 2008). Orthostatic dizziness may be associated with orthostatic hypotension that has prevalence 5–30%. However, most population-based studies showed dissociation between orthostatic dizziness and orthostatic hypotension (Ensrud et al., 1992; Colledge et al., 1996; Ooi et al., 1997; Wu et al., 2008). Up to 31% of elderly (Colledge et al., 1996) or nearly 20% of nursing home residents (Ooi et al., 1997) with dizziness or lightheadedness on standing do not have orthostatic blood pressure changes. Orthostatic dizziness is associated with age, medication use, and comorbid diseases, such as diabetes, stroke, vestibular dysfunction, vision impairment, disturbances in proprioception in addition to orthostatic blood pressure changes (Lipsitz, 1985; Mader et al., 1987; Sloane and Baloh, 1989; Sloane et al., 1989; Ensrud et al., 1992; Katsarkas, 1994). Orthostatic dizziness may be due to cerebral hypoperfusion (Wollner et al., 1979; Lipsitz, 1989; Sloane and Baloh, 1989; Sloane et al., 1989; Ohashi et al., 1991; Katsarkas, 1994; Novak et al., 1998). In most cases, the hemodynamic mechanisms leading to orthostatic dizziness without orthostatic hypotension are unknown.

**Figure 1:**
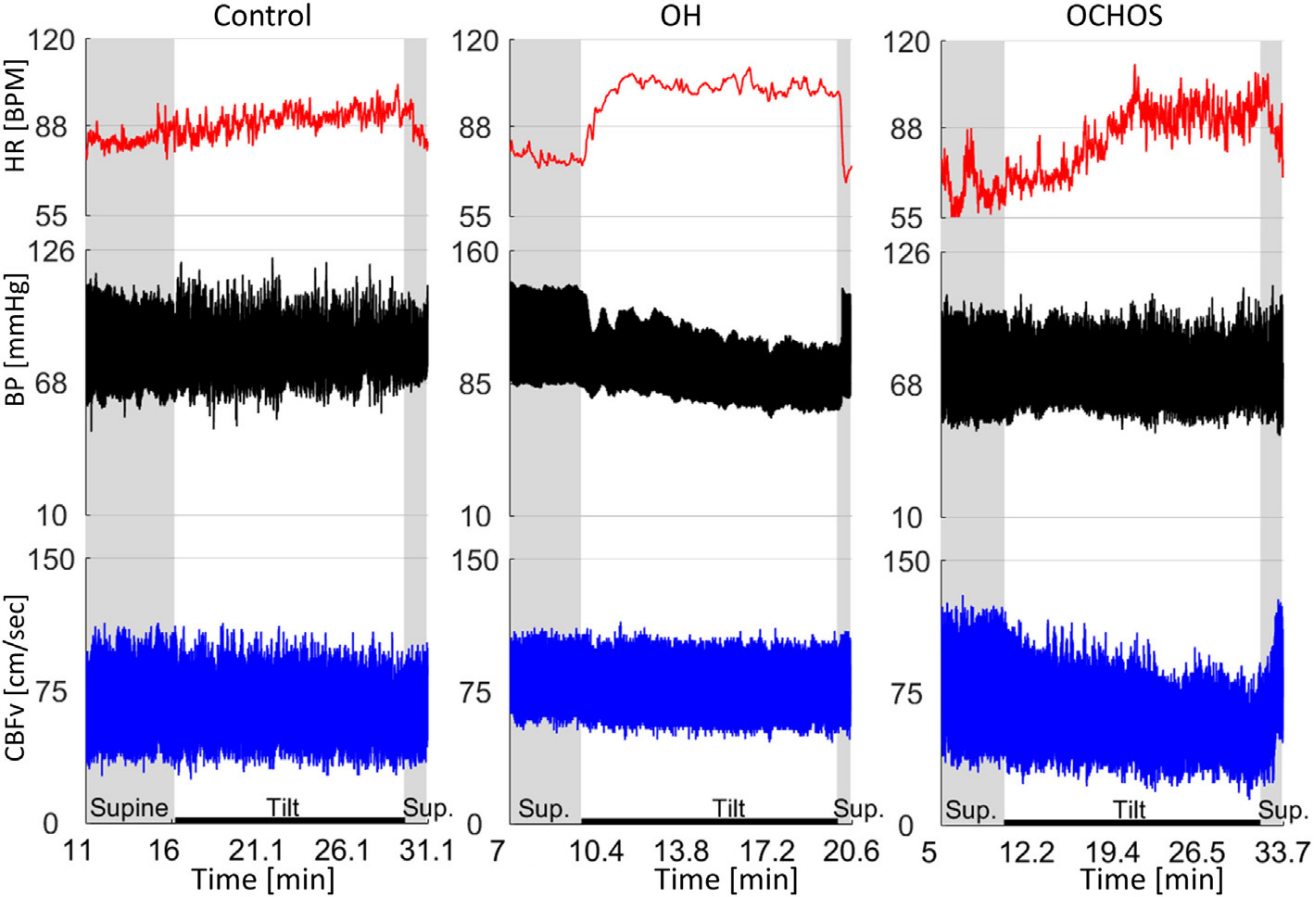
**Representative examples of normal orthostatic blood pressure and cerebral blood flow velocity (CBFv) (left panel), orthostatic hypotension (OH) with stable CBFv during tilt test (middle panel), and orthostatic cerebral hypoperfusion syndrome (OCHOs) (right panel)**. A patient with OH was asymptomatic and had stable CBFv during tilt test indicating preserved cerebral autoregulation. Patient with OCHOs had stable orthostatic blood pressure but reduced CBFv during the tilt test. He was dizzy during the tilt test. HR, heart rate; BP, blood pressure.

Recently, validated Quantitative Scale for Grading of Cardiovascular Autonomic Reflex Tests and Small Fibers from Skin Biopsies (QASAT) (Novak, 2015) grades abnormalities in orthostatic blood pressure in patients with autonomic failure that is typically associated with orthostatic symptoms, including dizziness. QASAT also evaluates cerebral orthostatic hypoperfusion by scoring cerebral blood flow velocity (CBFv) during the tilt test. The QASAT validation study showed that many subjects without orthostatic hypotension had reduced orthostatic CBFv [Fig. 1G and 1H in Novak (2015)]. This observation suggests that orthostatic cerebral hypoperfusion may exist without orthostatic hypotension in sizeable number of patients with orthostatic symptoms. This study follows the above observation and defines orthostatic cerebral hypoperfusion syndrome (OCHOs). Underlying hypothesis being tested is that OCHOs is a distinct syndrome of low CBFv but without orthostatic hypotension. The current study also combines the data used in the QASAT validation study (Novak, 2015) with more recent population expanding significantly number of evaluated patients.

## Materials and Methods

### Standard Protocol Approvals, Registrations, and Patient Consents

The study was approved by the Institutional Review Board of the University of Massachusetts Medical School as a minimal risk study and the consent form signature was waived.

### Study Population

This retrospective, single-center study included consecutive patients who underwent autonomic testing between 2008 and 2014 at the University of Massachusetts Medical School Autonomic laboratory. The inclusion criteria were: (1) history of orthostatic symptoms, including dizziness, weakness, fatigue, visual blurring, vertigo, suboccipital, paracervical or chest pain, headaches, dyspnea, palpitations, and low back pain (Low et al., 1995); (2) age 18 years or older, and (3) completion of the autonomic testing. Exclusion criteria were as follows: (1) history of significant arrhythmia, hypovolemia, endocrine disorder, or other metabolic derangement or systemic disease associated with autonomic failure.

Patient’s electronic records were reviewed for details about past medical history, laboratory evaluations, including blood work and, as well as the use of medication. The records were screened for history of autonomic failure, cardiac arrhythmia, and abnormal fluid volume. Neurological examinations were reviewed to identify abnormalities in sensory and autonomic evaluations. Particular emphasis was placed on identifying orthostatic hypotension, vestibular dysfunction, prominent vision, and proprioceptive impairment that can be associated with orthostatic dizziness (Lipsitz, 1985; Mader et al., 1987; Sloane and Baloh, 1989; Sloane et al., 1989; Ensrud et al., 1992; Katsarkas, 1994).

Medication that affects autonomic testing or causing orthostatic hypotension was stopped for five half-lives if this was considered to be safe and discontinuation was tolerated by patients.

### Autonomic Testing

All testing was performed following established standards (Low, 1993; Novak, 2011) and was described in details previously (Novak, 2011). Cardiovascular reflex tests included deep breathing, the Valsalva maneuver, and the tilt test. Patients were tilted at 70° for 10 min or more following 10 min of supine rest. Recorded signals include electrocardiogram, blood pressure, respiratory movement using a nasal thermistor, end tidal CO_2_ using Capstar-100 (CWE, Inc., Ardmore, PA, USA) and CBFv in the middle cerebral artery using Transcranial Doppler. Baseline supine blood pressure was obtained intermittently using an automated oscillometric blood pressure device Dinamap ProCare Monitor 100 (GE, Fairfield, CT) from arm and continuously (beat-to-beat) using Finometer^®^ (Finapress Medical Systems, Amsterdam, Netherlands) from the third finger. During the tilt test, blood pressure was obtained every minute using the Dinamap ProCare device and continuously using Finometer^®^. The Dinamap obtained blood pressure served as a reference for the Finometer-based blood pressure. Finometer-based blood pressure was calibrated with the Dinamap-based blood pressure.

The temporal acoustic window with a 2 MHz probe was used for acquisition of CBFv using a MultiDop T (DWL, Singen, Germany). The left middle cerebral artery has been identified at an insonation depth from 45 to 65 mm. After the optimal flow signal has been detected, transducer has been attached to head with a plastic head frame with a three-dimensional positioner to maintain a tight transducer fixation at constant depth and angle of insonation. Continuous Trancranial Doppler monitoring has been performed during the supine baseline period and the tilt. Signals were recorded using LabChart 7 system (ADInstruments Inc., Colorado Springs, CO, USA) and sampled at 400 Hz.

Normative data for CBFv at both supine and tilt test are age, gender, and the tilt duration dependent. In healthy subjects, the CBFv is either unchanged or there can be a mild drop in CBFv during the tilt. The criteria for normal drop of the mean CBFv during tilt test are equal to 90/% (1st minute), 89% (5th minute), and 85% (10th minute) of the tilt where baseline is equal to 100% (Novak, 2015).

Instantaneous heart rate, systolic, mean, diastolic blood pressure, and CBFv were obtained on beat-to-beat basis. Resistance index (RI) and cerebral vascular resistance (Novak et al., 1998) were also calculated. RI was defined as (systolic CBFv–diastolic CBFv)/systolic CBFv and cerebral vascular resistance was defined as mean blood pressure/mean CBFv.

The results of autonomic testing were graded using QASAT (Novak, 2015). QASAT is an objective and validated instrument that grades cardiovascular reflex tests, including CBFv responses to tilt test. QASAT has the following sections: heart rate (includes supine heart rate, heart rate response to tilt, and heart rate variability), blood pressure (supine and tilt-induced changes, including orthostatic hypotension/hypertension), cerebral blood flow (both baseline and tilt-induced changes), sudomotor, and sensory. QASAT assigns a numeric value to each test result that is proportional to the severity of findings, 0 = normal, >0 abnormal. Higher scores indicate more severe impairment. The QASAT criteria were solved by genetic algorithms (Forrest, 1993) mimicking process of natural selection and evolution.

The heart rate and blood pressure were analyzed at the supine baseline immediately before tilt, and every minute during tilt. CBFv, RIs, and end tidal CO_2_ were analyzed at the supine baseline and at the 1st, 5th, and 10th minute of tilt as required by QASAT.

All subjects were observed for presence of orthostatic symptoms during tilt test. Orthostatic symptoms were defined as: (1) symptoms that occur during the upright position and are relieved at supine and (2) symptoms should be present without any body movement, both in the supine and upright position. The symptoms include dizziness, lightheadedness, visual disturbances, hearing disturbances, vertigo, fatigue, sweating, weakness, headache, neck pain, chest pain, palpitations, confusion, agitation, memory problems, and inattentiveness (Task Force for the Diagnosis and Management of Syncope et al., 2009). The symptoms occurring during the movement of the tilt table were considered non-orthostatic even if they persisted after secession of the tilt table movement.

### OCHOs Definition

Orthostatic cerebral hypoperfusion syndrome was defined by the following inclusion and exclusion criteria. Inclusion criteria were (1) abnormal orthostatic drop of mean CBFv fulfilling criteria of at least moderate abnormality on the QASAT-CBF (section 15 > 2, Table 1) obtained at the 1st, 5th, and 10th minute of the tilt.

**Table 1:** **Cerebral blood flow category of QASAT**.

Results	Grading	Value	Definition^a^
15. Blood flow response to tilt
Normal		0
	Mild	1,2
Abnormal	Moderate	3–4	
	Severe	>4	

*Using mean CBF_V_. CBF_V_ should be measured at baseline, and at 1, 5, and 10 min of tilt*.

*^a^At each measurement, assign the value 0 for normal results. Abnormal results are defined as CBF_V_ less than normal limit. Normal limit is defined as follows*:

*At 1 min: normal decline in CBF_V_ ≥ 90% of baseline. Assign 1 for CBF_V_ 80–89% of baseline, 2 for 70–79% of baseline, and 3 for <70% of baseline*.

*At 5 min: normal decline in CBF_V_ ≥ 89% of baseline. Assign 1 for CBF_V_ 79–88% of baseline, 2 for 69–78% of baseline, and 3 for <69% of baseline*.

*At 10 min: normal decline in CBF_V_ ≥ 86% of baseline. Assign 1 for CBF_V_ 77–85% of baseline, 2 for 67–76% of baseline, and 3 for <67% of baseline. Total score is obtained by sum of all scores. Revised after (Novak, 2015)*.

Exclusion criteria include (1) presence of orthostatic hypotension (QASAT section 7 > 0); (2) presence of arrhythmia defined as presence of bradycardia during supine or tilt test (QASAT section 1 > 0) or tachycardia during the tilt test (QASAT section 3 > 1). This criterion also excludes postural tachycardia syndrome (POTS) (Novak et al., 1998); (3) unable to complete the 10 min of tilt test for any reason including syncope; (4) evidence of hyperventilation during tilt test [end tidal CO_2_ < 35 mm Hg (Ocon et al., 2009)]; (5) significant structural abnormality on brain imaging that can cause significant hemodynamic abnormalities using CT or MRI of the brain; (6) any other cause of abnormal intracranial velocities, including cerebrovascular accident affecting large vessels, abnormal hematocrit, or volemic status; (7) deficit in sensory systems, including vestibular, visual, or proprioceptive impairment that can results in orthostatic dizziness; (8) the use of medication that affect autonomic functions, including vasoactive medication (vasodilatators or vasoconstrictors) during testing. These medications were as follows: any drug for treatment of hypertension, including calcium channel blockers, beta blockers, nitrates, diuretics, angiotensin-converting enzyme inhibitors, anticholinergics, fludrocortisone, pyridostigmine, and proamatine.

To satisfy the OCHOs criteria, all inclusion and none of exclusion criteria has to be met.

### Control Subjects

The OCHOs subjects were matched with a control group by age, gender, and body mass index from our Autonomic database at University of Massachusetts. All controls had normal response to tilt in heart rate, blood pressure, and CBFv and respiratory variables.

### Statistical Analysis

One-way analysis of variance (ANOVA) was used to test differences between the OCHOs and non-OCHOs patients at baseline, 1st minute, 5th minute, and 10th minute of tilt. The initial significance level (α) has been set to 0.05. Since there were four comparisons, the Bonferroni-corrected significance level was adjusted to 0.0125 (obtained by 0.05/4). The effect of tilt within group was analyzed by MANOVA for repeated measures. JMP 12 (Cary, NC, USA) statistical software was used for all statistical analyses.

## Results

From 1279 patients who were referred for evaluation of unexplained orthostatic dizziness, 102 patients satisfied criteria for diagnosis of OCHOs. These patients were age- and gender-matched with the same number of control subjects with normal responses to tilt. Table 2 shows basic demographic of OCHOs group. The following comorbidities were found in OCHOs patients: hypertension [*n*, disease duration (years, mean ± SD)] 21, 5.9 ± 3.4, migraine 14, 5.1 ± 3.4, bipolar disorder (14, 4.15 ± 0.9), polyneuropathy (11, 10.4 ± 9.7), dyslipidemia (10, 4.4 ± 1.4), diabetes (9, 3.6 ± 1.6), fibromyalgia (7, 3.8 ± 1.4), autonomic neuropathy (5, 4.75 ± 4.3), Parkinson disease (5, 4.75 ± 2.62), hypothyroidism (4, 4.25 ± 2.1), chronic fatigue syndrome (3, 6.3 ± 4.9), anxiety (3, 4.6 ± 0.5), and connective tissue disorder (2, 4.5 ± 0.7).

**Table 2:** **Demographic data**.

Variable	Controls	OCHOs	P
Number of participants, f/m	102,60/42	102,60/42	1
Age, years, range	49.9 ± 15.9, 18–85	51.1 ± 14.9, 19–84	0.587
BMI (kg/m^2^)	27.8 ± 6.5	27.6 ± 6.0	0.768

*OCHOs, orthostatic cerebral hypoperfusion syndrome*.

**Figure 2:**
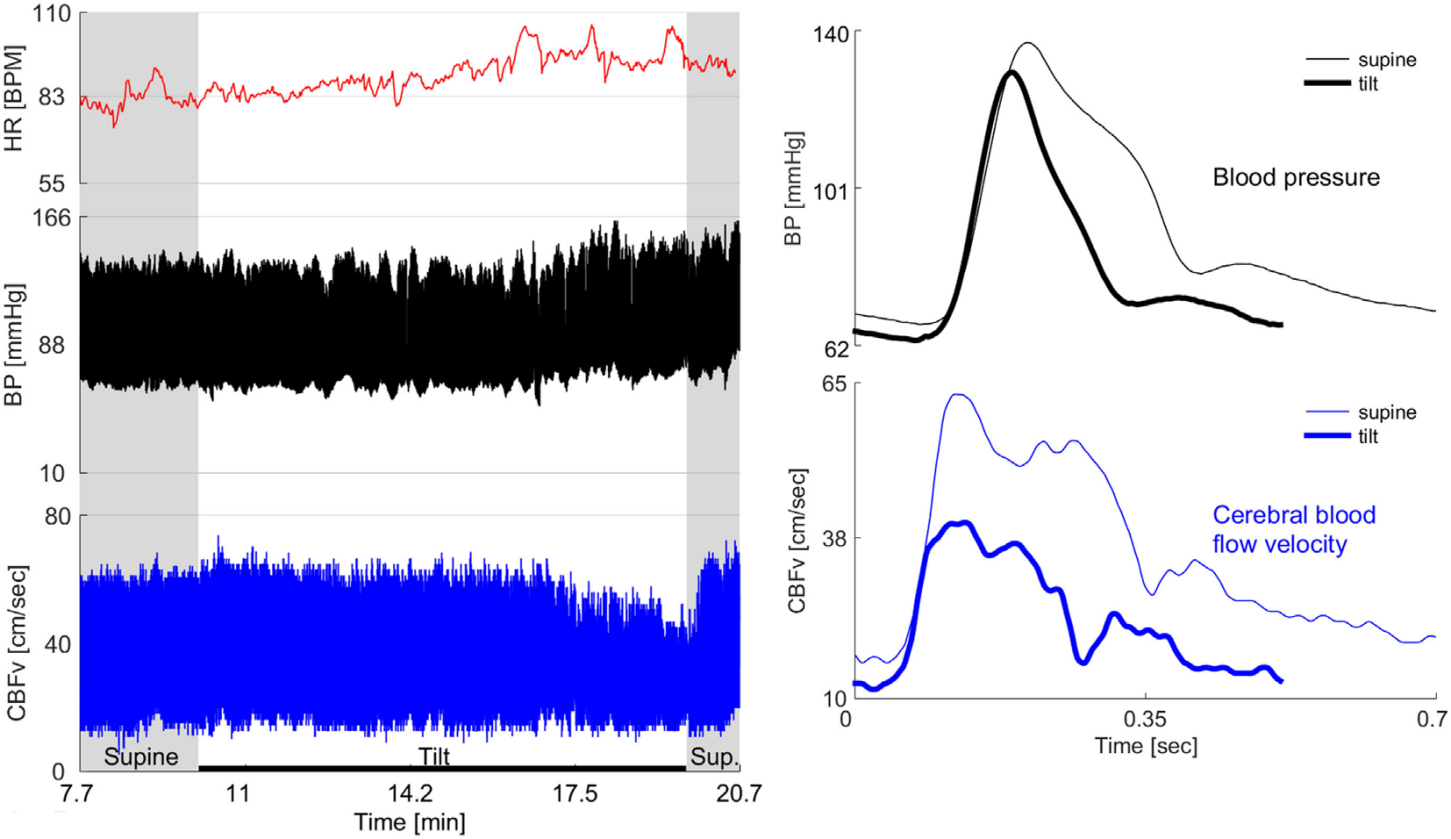
**Example of severe OCHOs**. 72-year-old man had diabetes for 2 years and hypertension for 12 years. For last year, he experienced orthostatic dizziness and multiple presyncopal episodes. He was not orthostatic at the office visits and psychogenic or cardiac cause of orthostatic symptoms has been suspected. Left panel shows the baseline supine period before and after the tilt and the tilt responses. Right panel shows one cardiac cycle of blood pressure and cerebral blood flow velocity (CBFv) at the supine baseline and at the end of the tilt. The baseline CBFv gradually declined during tilt test. After the 5th minute of tilt, patient became progressively dizzy, confused, and disoriented that coincided with drop of CBFv. He recovered shortly after returning to the supine position when also the CBFv returned to the baseline value. There was no evidence cardiac dysrhythmia throughout the testing. There was no orthostatic hypotension during the tilt test, in fact, the blood pressure was elevated at the second half of the tilt that corresponds to a period when patient experienced orthostatic symptoms. HR, heart rate; BP, blood pressure.

Figure 1 shows a representative example of OCHOs with stable orthostatic blood pressure, preserved heart rate responses, and reduced CBFv during tilt. An example of severe OCHOs is on Figure 2. All OCHOs subjects were symptomatic during tilt test and many subjects experienced more than one symptom. Tilt-induced symptoms include dizziness or lightheadedness (88%), confusion (2%), headache (10%), tiredness (18%), anxiety (23%), generalized weakness (14%), vision changes (4%), leg heaviness (15%), and burning sensation at distal legs (12%).

### Baseline Supine Data

Comparing OCHOs and controls, there was no difference in all tested variables during the supine period before the tilt (Table 2). Both groups had equal blood pressure, heart rate, CBFv, RI, and cerebrovascular resistance (CVR).

### Response to Tilt

**Figure 3:**
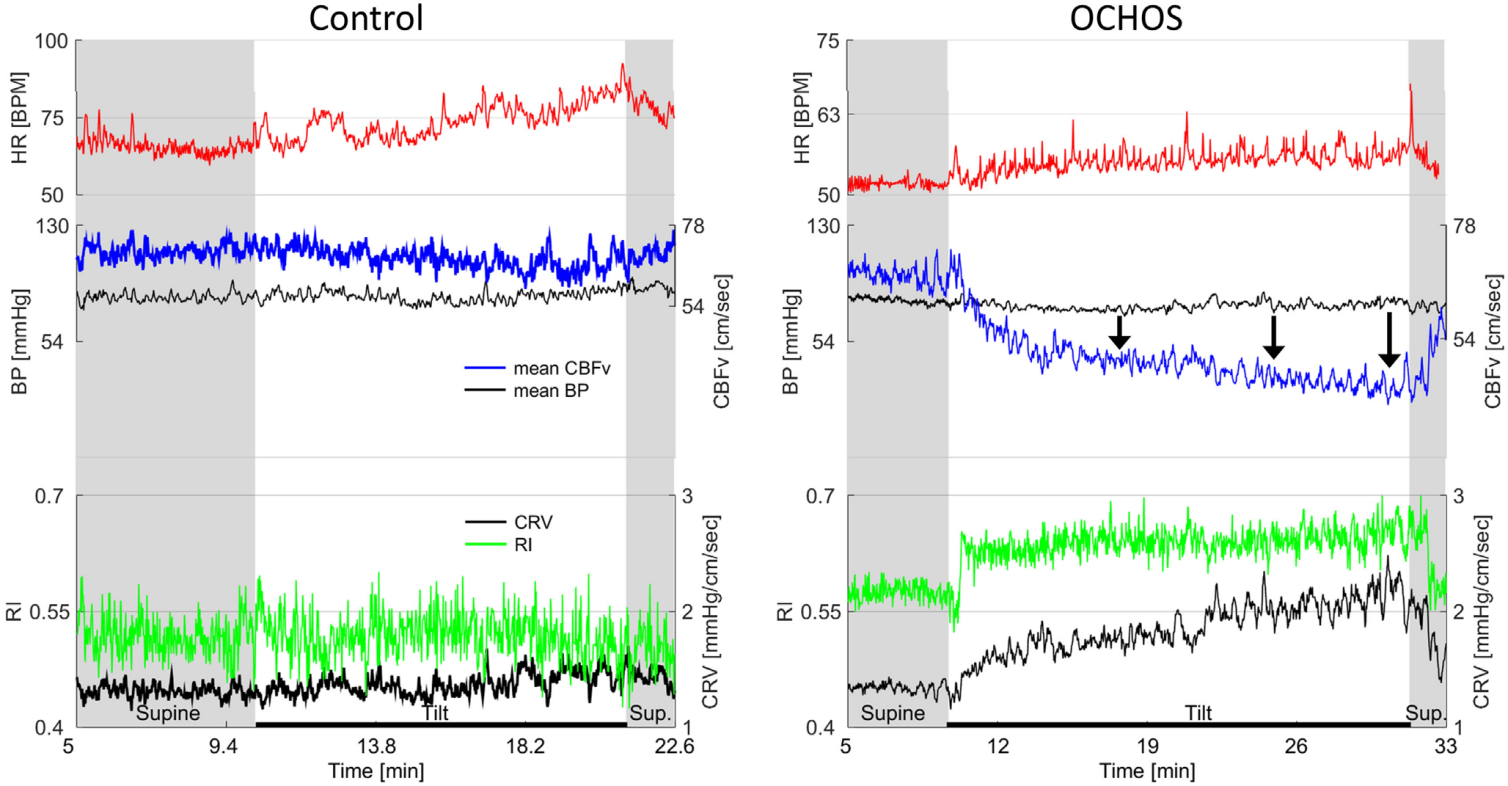
**Details of cerebral blood flow velocity (CBFv) in OCHOs (right) and in a control subject with normal response to tilt (left)**. OCHOs subject is 61-year-old man with history of orthostatic dizziness and neuropathy. During tilt, patient complained on dizziness with headache and he became very tired at the end of the tilt. Note progressive decline in CBFv during the tilt pointed by black arrows. Both resistance index (RI) and cerebrovascular resistance (CVR) increased in OCHOs while they remained unchanged in control subject during the tilt. The mean blood pressure was stable in both control and OCHOs subject during tilt. The heart responses to tilt were preserved in both subjects.

In most subjects, there was a progressive drop of systolic, mean, and diastolic CBFv’s in both groups during the tilt test (Table 3; Figures 1–3). In several OCHOs subjects, the CBFv fluctuated throughout the tilt with partial recovery. The drop in CBFv was markedly higher in OCHOs group. Mean CBFv was reduced to 75.6 ± 8.2% of baseline value that is equal to 24.4% reduction at the 10th minute of the tilt. The reduction of CBFv was equal to 95.8 ± 5.6% (4.2%) in control group at the 10th minute of the tilt. The difference between OCHOs and controls in CBFv drop was highly significant (*p* < 0.0001, Table 2). RI and cerebral vascular resistance were elevated throughout the tilt test in OCHOs group compared to controls. There was no difference in blood pressure and heart rate during the tilt in both groups. There was no difference in end tidal CO_2_ during baseline supine and during the tilt compared both groups.

**Table 3:** **Clinical variables**.

Variable	Controls	OCHOs	*P*
SCBFv at supine, cm/s (100%)	104.2 ± 18.1	104.3 ± 19.7	0.974
SCBFv drop from supine at 1st minute of tilt, % of baseline	96.8 ± 5.0	86.5 ± 8.8	<0.0001
SCBFv drop from supine at 5th minute of tilt, % of baseline	96.7 ± 6.3	81.8 ± 8.1	<0.0001
SCBFv drop from supine at 10th minute of tilt, % of baseline	95.3 ± 5.0	77.5 ± 7.8	<0.0001
MCBFv at supine, cm/s (100%)	65.1 ± 10.9	65.1 ± 11.8	0.967
MCBFv drop from supine at 1st minute of tilt, % of baseline	97.9 ± 4.5	84.8 ± 8.9	<0.0001
MCBFv drop from supine at 5th minute of tilt, % of baseline	98.1 ± 6.6	79.6 ± 8.6	<0.0001
MCBFv drop from supine at 10th minute of tilt, % of baseline	95.8 ± 5.6	75.9 ± 8.2	<0.0001
DCBFv at supine, cm/s (100%)	45.5 ± 9.5	45.3 ± 9.7	0.879
DCBFv drop from supine at 1st minute of tilt, % of baseline	99.4 ± 7.6	83.3 ± 13.1	<0.0001
DCBFv drop from supine at 5th minute of tilt, % of baseline	98.8 ± 8.2	77.9 ± 13.1	<0.0001
DCBFv drop from supine at 10th minute of tilt, % of baseline	96.5 ± 8.7	73.7 ± 12.5	<0.0001
SBP supine, mmHg	122.9 ± 15.1	122.6 ± 14.6	0.895
SBP at first minute of tilt, mmHg	123.2 ± 15.4	122.2 ± 14.7	0.643
SBP at fifth minute of tilt, mmHg	122.6 ± 15.4	121.3 ± 14.9	0.532
SBP at 10th minute of tilt, mmHg	121.6 ± 13.9	119.9 ± 14.8	0.412
MBP supine, mmHg	91.1 ± 9.4	90.5 ± 10.6	0.620
MBP at 1st minute of tilt, mmHg	93.2 ± 10.1	93.3 ± 10.4	0.995
MBP at 5th minute of tilt, mmHg	93.2 ± 10.5	91.9 ± 9.7	0.365
MBP at 10th minute of tilt, mmHg	92.1 ± 9.6	90.4 ± 9.7	0.200
DBP supine, mm Hg	75.2 ± 7.6	74.5 ± 9.8	0.560
DBP at 1st minute of tilt, mmHg	78.3 ± 8.5	78.4 ± 8.9	0.923
DBP at 5th minute of tilt, mmHg	78.2 ± 9.3	77.1 ± 8.7	0.391
DBP at 10th minute of tilt, mmHg	77.1 ± 8.40	75.5 ± 8.45	0.187
HR supine, BPM	71.8 ± 11.9	72.5 ± 11.8	0.672
HR at 1st minute of tilt, BPM	78.6 ± 13.2	81.0 ± 13.9	0.209
HR at 5th minute of tilt, BPM	81.3 ± 12.5	84.0 ± 13.9	0.144
HR at 10th minute of tilt, BPM	82.5 ± 12.5	85.8 ± 15.2	0.09
End tidal CO_2_ supine, mmHg	40.5 ± 2.70	40.1 ± 2.44	0.45
End tidal CO_2_ at 1st minute of tilt, mmHg	39.5 ± 2.01	39.4 ± 2.1	0.714
End tidal CO_2_ at 5th minute of tilt, mmHg	38.9 ± 2.05	38.9 ± 2.1	0.985
End tidal CO_2_ at 10th minute of tilt, mmHg	38.7 ± 1.15	38.5 ± 2.1	0.614
Resistance index supine	0.56 ± 0.08	0.56 ± 0.08	0.909
Resistance index at 1st minute of tilt	0.54 ± 0.08	0.57 ± 0.08	0.0045
Resistance index at 5th minute of tilt	0.54 ± 0.08	0.58 ± 0.08	0.0029
Resistance index at 10th minute of tilt	0.55 ± 0.01	0.58 ± 0.09	0.01
CVR supine, mmHg/cm/s	1.44 ± 0.31	1.45 ± 0.31	0.861
CVR at 1st minute of tilt, mmHg/cm/s	1.49 ± 0.31	1.76 ± 0.40	<0.0001
CVR at 5th minute of tilt, mmHg/cm/s	1.50 ± 0.30	1.85 ± 0.45	<0.0001
CVR at 10th minute of tilt, mmHg/cm/s	1.52 ± 0.32	1.92 ± 0.43	<0.0001

*Data are mean ± SD. Percentages are expressed as percent of baseline where baseline is equal to 100%. OCHOs, orthostatic cerebral hypoperfusion syndrome; SCBFv/MCBFv/DCBFv, systolic/mean/diastolic cerebral blood flow velocity; SBP/MBD/DBP, systolic/mean/diastolic blood pressure; HR, heart rate, CVR, cerebral vascular resistance*.

MANOVA for repeated measures showed significant effect of tilt upon end tidal CO_2_ (*p* < 0.0001), mean CBFv (*p* < 0.0001), and heart rate (*p* < 0.0001) but not on systolic/mean/diastolic blood pressure at *p* = 0.05 level at both groups.

All subjects with OCHOs and none of the control subjects had orthostatic symptoms during the tilt test.

## Discussion

Orthostatic cerebral hypoperfusion syndrome is a novel syndrome associated with orthostatic hypoperfusion. OCHOs patients have stable orthostatic blood pressure, heart rate, and respiratory pattern and they still have reduced orthostatic CBFv. A decrease in orthostatic CBFv may produce cerebral hypoperfusion and central nervous system symptoms, including dizziness (Wollner et al., 1979; Ohashi et al., 1991; Novak et al., 1998; Ocon et al., 2009).

Other syndromes that are associated with orthostatic dizziness or orthostatic intolerance without orthostatic hypotension were ruled out in this study. These syndromes include orthostatic tachycardia syndrome (Low et al., 2009), inappropriate sinus tachycardia (Yusuf and Camm, 2005), large cerebral vessel disease (Ouchi et al., 2001), and deficit in sensory systems, including vestibular dysfunction, vision impairment, or disorders in the proprioceptive system (Wu et al., 2008). POTS and syncope patients were removed from this study by exclusion criteria. There was no evidence of significant sensory deficit in our patients that could account for orthostatic dizziness. All OCHOs patients had either CT or MRI of the brain and no one had evidence of large structural lesion or evidence of cerebral large vessel disease.

Transcranial Doppler measures flow velocity instead of blood flow. The velocity is not only proportional to flow but also depends on the diameter of the insonated vessel. The MCA diameter is resistant to change during orthostatic stress (Serrador et al., 2000) and, therefore, CBFv is considered a good surrogate of cerebral blood flow (Medow et al., 2014).

OCHOs patients had reduced mean CBFv by more than 20% compared to controls. That reduction is sufficient to induce symptoms of cerebral hypoperfusion. Reduction in CBFv by 18.7% (Novak et al., 1998) and 19.5% (Ocon et al., 2009) from the supine baseline period is associated with signs of central nervous system dysfunction in POTS patients. Tilt-induced CBFv dropped by 26% was observed at the time of loss of consciousness in cerebral syncope patients (Grubb et al., 1998). In healthy subjects, the drop of CBFv below 30% of the baseline is associated with signs of cerebral hypoperfusion and drop by 50% of the baseline is associated with syncope (Njemanze, 1992).

The criteria for the CBFv changes in OCHOs were derived from QASAT. QASAT grades tilt-induced CBFv abnormalities into mild–moderate–severe. Moderate CBFv abnormalities were chosen as a threshold, since this level of abnormality segregate patients with more widespread dysautonomia as seen in diabetes and Parkinson disease from mild CBFv abnormalities as seen in non-diabetic autonomic neuropathies (Novak, 2015). If stricter criteria are used, for example, severe abnormalities on the QASAT-CBF, then 49 patients would still satisfy the OCHOs criteria. The grading criteria are time dependent. Severe CBFv abnormalities are defined as presence at least two out three of the following: the drop of CBFv below 70% at the 1st minute, 69% at the 5th minute, or 67% at the 10th minute where the baseline is equal to 100%.

Orthostatic cerebral hypoperfusion syndrome affects wide range of age groups and more women than men were affected. However, this could be due referral bias and needs to be confirmed in subsequent studies. Several comorbidities has been found in OCHOs, hypertension being the most frequent (21%). Whether this is real association between hypertension and OCHOs or it reflects referral bias needs to be assessed in further studies.

There are several mechanisms that may lead to cerebral hypoperfusion in OCHOs. Cerebral perfusion is controlled by a complex system called cerebral autoregulation that determines relationship between CBFv and the blood pressure. CBFv is kept constant within relatively large variations of the blood pressure by adjusting the vascular resistance. Static autoregulation determines the average CBFv at a given blood pressure under steady-state conditions. Then low CBFv with stable orthostatic blood pressure and heart rate indicates failure of cerebral autoregulation such as the static cerebral autoregulation is reduced. The mechanism associated with the autoregulatory failure in OCHOs can be abnormal vasoconstriction at the cerebral arterioles that reduces the cerebral flow. Cerebral vasoconstriction was proposed as underlying mechanism for reduced orthostatic CBFv in healthy subjects and in orthostatic tachycardia syndrome (Dewey et al., 1974; Njemanze, 1992; Franco Folino, 2007; van Beek et al., 2008; Tzeng and Ainslie, 2014). Either sympathetic-mediated cerebral vasoconstriction (Immink et al., 2006) or parasympathetic withdrawal-mediated reduction of nitric-oxide-dependent cerebral vasodilatation (Del Pozzi et al., 2014) were proposed. Both mechanisms involve active processes leading to cerebral vasoconstriction.

Another mechanism leading to OCHOs may be related to ineffective compensation to orthostatic stress. Changing of position to standing reduces central blood volume with redistribution to the splanchnic vasculature and lower limbs (Del Pozzi et al., 2014). It can be hypothesized that excessive reduction of central blood volume due to increased venous compliance that reduces venous return may reduce cardiac output in OCHOs. The sympathetic activation to orthostatic stress still can compensate for reduced cardiac output by increasing sympathetically mediated peripheral resistance and, hence, to maintain the orthostatic blood pressure, but not enough to maintain the orthostatic cerebral flow.

Both OCHOs and some POTS patients share orthostatic drop of CBFv without orthostatic hypotension. In contrast to POTS, orthostatic tachycardia and orthostatic hyperventilation is not feature of OCHOs, hence different mechanisms may play a role. Nevertheless, it is possible that a normocapnic POTS (Ocon et al., 2009) is a form of OCHOs with exaggerated heart rate response to tilt. Cerebral syncope (Njemanze, 1993; Daffertshofer and Hennerici, 1995; Fredman et al., 1995; Grubb et al., 1998) also shares similarities with OCHOs. In both syndromes, the orthostatic CBFv drops without associated orthostatic hypotension or bradycardia during the tilt test. Nevertheless, OCHOs fundamentally differs from cerebral syncope. The systolic and the diastolic CBFv drops in OCHOs indicating either cerebral vasoconstriction or a passive loss of intravascular volume (see the discussion above). In cerebral syncope, the systolic CBFv is elevated and the diastolic CBFv is reduced (Grubb et al., 1998), e.g., the pulsatility index (systolic–diastolic CFBv) increases that is consistent with cerebral vasodilation (Carey et al., 2002; Ocon et al., 2011).

The two most common comorbidities in OCHOs patients were hypertension and migraine, combined they represent 35% of all OCHOs patients. Both disorders are associated with small vessel disease (SVD) (Agostoni and Rigamonti, 2012; Mok and Kim, 2015). If combining all disorders found in OCHOs which are associated with SVD (hypertension, migraine, diabetes, and dyslipidemia), then total 52% of OCHOs patients (some of the patients have more than one risk factor) have risk factors for SVD. Hence, SVD represents possible link of vascular pathology to OCHOs. Nevertheless, this needs to be investigated in further studies. Medication is unlikely responsible for results since patients taking vasoactive medication were excluded from the study.

This study defines the OCHOs as a novel syndrome associated with low orthostatic CBFv and stable orthostatic blood pressure, heart rate, and respiratory pattern. OCHO, thus, complements common orthostatic hemodynamic syndromes, including POTS (principal abnormality in heart rate), OH (principal abnormality in blood pressure), and OCHOs (principal abnormality in cerebral blood flow). Using an orthostatic triad (POTS, OH, and OCHOs) classification can set up a useful clinical framework for evaluation of orthostatic symptoms. To evaluate patients using the outlined classification, it is essential to monitor CBFv in addition to heart rate, blood pressure, and respiratory variables during the tilt test.

Results presented in this paper may have direct clinical significance. The concept of OCHOs provides physiologically plausible mechanism for unexplained orthostatic dizziness that is very common. The proposed concept of OCHOs relies on inexpensive technology (transcranial Doppler, heart rate, and blood pressure monitors) and normative data are provided.

This study has several limitations. The principal limitation is its retrospective character where the OCHOs criteria were applied to already acquired data and the exact criteria may not be applicable to other population. Second, a referral bias may affect selection of subjects and, therefore, the studied population may not be representative. Third, a bias could be introduced by using the particular transcranial Doppler device since the CBFv values may be device dependent (Novak, 2015). Fourth, all CBFv’s were acquired by one sonographer (Peter Novak) that was not blinded to evaluations. There are potential other biases associated with a single-center, retrospective study. Only a prospective study, preferentially blinded to a sonographer, can resolve these limitations.

In conclusion, OCHOs may be a common cause of orthostatic dizziness in patients without orthostatic hypotension. Orthostatic dizziness in OCHOs is consistent with central nervous system hypoperfusion due to low orthostatic CBFv with a failure of static cerebral autoregulation most likely due excessive cerebral vasoconstriction upon upright posture. Detection of OCHOs is straightforward by simultaneous monitoring of hemodynamic variables and CBFv during the tilt test.

## Author Contributions

PN designed the study, participated in data collection, performed data analysis, and wrote the manuscript.

## Conflict of Interest Statement

The author declares that the research was conducted in the absence of any commercial or financial relationships that could be construed as a potential conflict of interest. The reviewers, TP and BM, and handling Editor declared their shared affiliation, and the handling Editor states that the process nevertheless met the standards of a fair and objective review.

## Abbreviations

BP, blood pressure; CBFv, cerebral blood flow velocity; HR, heart rate; OH, orthostatic hypotension, OCHOs, orthostatic cerebral hypoperfusion syndrome; POTS, postural tachycardia syndrome; QASAT, quantitative scale for grading of cardiovascular autonomic reflex tests and small fibers from skin biopsies; SVD, small vessel disease.
